# Subsurface imaging of flexible circuits via contact resonance atomic force microscopy

**DOI:** 10.3762/bjnano.10.159

**Published:** 2019-08-07

**Authors:** Wenting Wang, Chengfu Ma, Yuhang Chen, Lei Zheng, Huarong Liu, Jiaru Chu

**Affiliations:** 1 Department of Precision Machinery and Precision Instrumentation, University of Science and Technology of China, Hefei 230026, Anhui, China; 2 The 38th Research Institute of China Electronics Technology Group Corporation, Hefei 230088, Anhui, China

**Keywords:** atomic force microscopy (AFM), contact resonance atomic force microscopy (CR-AFM), contact stiffness, defect detection, flexible circuits, subsurface imaging

## Abstract

Subsurface imaging of Au circuit structures embedded in poly(methyl methacrylate) (PMMA) thin films with a cover thickness ranging from 52 to 653 nm was carried out by using contact resonance atomic force microscopy (CR-AFM). The mechanical difference of the embedded metal layer leads to an obvious CR-AFM frequency shift and therefore its unambiguous differentiation from the polymer matrix. The contact stiffness contrast, determined from the tracked frequency images, was employed for quantitative evaluation. The influence of various parameter settings and sample properties was systematically investigated by combining experimental results with theoretical analysis from finite element simulations. The results show that imaging with a softer cantilever and a lower eigenmode will improve the subsurface contrast. The experimental results and theoretical calculations provide a guide to optimizing parameter settings for the nondestructive diagnosis of flexible circuits. Defect detection of the embedded circuit pattern was also carried out, which indicates the capability of imaging tiny subsurface structures smaller than 100 nm by using CR-AFM.

## Introduction

With the rapid shrinkage of microelectronic devices, flexible circuits are intensively used while being functionalized as supercapacitors [1–4], heaters [5–8] and temperature sensors [9–12]. Successful applications can be found in smart contact lenses, transparent electronic devices and deformable electronic skin, for instance. In general, a flexible circuit consists of a highly flexible thin polymer film as the substrate on which conductive metal circuits are patterned. Another thin polymer coating is employed to protect the circuits from contamination and short circuiting. Such a sandwich structure permits the device to bend and fold flexibly, yet with other advantages including small volume, light weight, and so forth. However, defects and fractures may emerge during either the fabrication or with repeated usage. As a result, detecting buried structures in the circuits with high spatial resolution is of critical importance. Traditional optical imaging is on the one hand not applicable for opaque cover layers and on the other hand the resolution is quite limited. Cross-sectional approaches can provide through-depth information, yet they are intrinsically destructive and require complicated sample processing. To meet such challenges, noninvasive subsurface imaging based on scanning probe microscopy (SPM) has emerged as a promising way.

Various SPM-based nanoscale subsurface imaging methods have been proposed that rely on different detection mechanisms including thermal, magnetic, electric, and mechanical sensing. Among them, contact resonance atomic force microscopy (CR-AFM) demonstrates the unique advantages of easy operation and no special requirements for the tip and sample. In this method, the tip–sample contact is modulated with ultrasonic vibrations and the contact resonance of the AFM probe is monitored while scanning in contact mode. CR-AFM was started from the so-called atomic force acoustic microscopy where the sample is ultrasonically excited at a specified frequency and the amplitude and phase of the cantilever are recorded [13–14]. After that, the theoretical analysis of the cantilever vibration with the tip contacting the sample surface was intensively investigated, including the influence of tip position on the cantilever, lateral forces and cantilever tilt [14–16]. Since the contact resonance is sensitive to the sample’s local mechanical properties, CR-AFM has been employed for characterization of elastic and viscoelastic properties [17–20]. In addition, mechanically heterogeneous structures in the contact volume will alter the local contact stiffness and then the contact resonance of the cantilever. Its usage in detecting buried structures such as defects [21–25] and nanofillers [26–28] has thus gained much attention. Although a few investigations have been carried out using CR-AFM for subsurface imaging, its application in defect diagnosis for flexible circuits has seldom been reported. Intuitively, the presence of buried metal circuit patterns in the tip-generated stress field will alter the local indentation modulus. This consequently leads to frequency shifts of the contact resonances. Therefore, CR-AFM is expected to also possess the ability of nondestructively detecting the buried circuit structures. Nevertheless, a systematic investigation on the influences of various experimental parameters is still of critical importance to enable unambiguous subsurface imaging.

In this work, facing the demands and challenges mentioned above, CR-AFM subsurface imaging was performed on a series of multilayer flexible circuits. Model samples were employed that consist of Au circuit patterns embedded in a poly(methyl methacrylate) (PMMA) polymer matrix. The influence of some key imaging factors was investigated including the applied normal force, cantilever stiffness, vibration eigenmode, and the elastic properties and thickness of each layer. The experimental results were then interpreted with theoretical analysis considering the dynamic model of the cantilever and the contact mechanics between the tip and the multilayer sample. Finite element analysis (FEA) was also carried out for comparison. Some qualitative clues were obtained for optimizing the imaging contrast, which were experimentally proved. Finally, imaging of structural defects in the buried circuit patterns was exhibited. Our work shows that CR-AFM can be used to detect embedded micrometer- and nanometer-scale circuits and their defects, and quantitative analysis of contact stiffness contrast can be achieved.

## Methods

### Sample preparation

**Figure 1:**
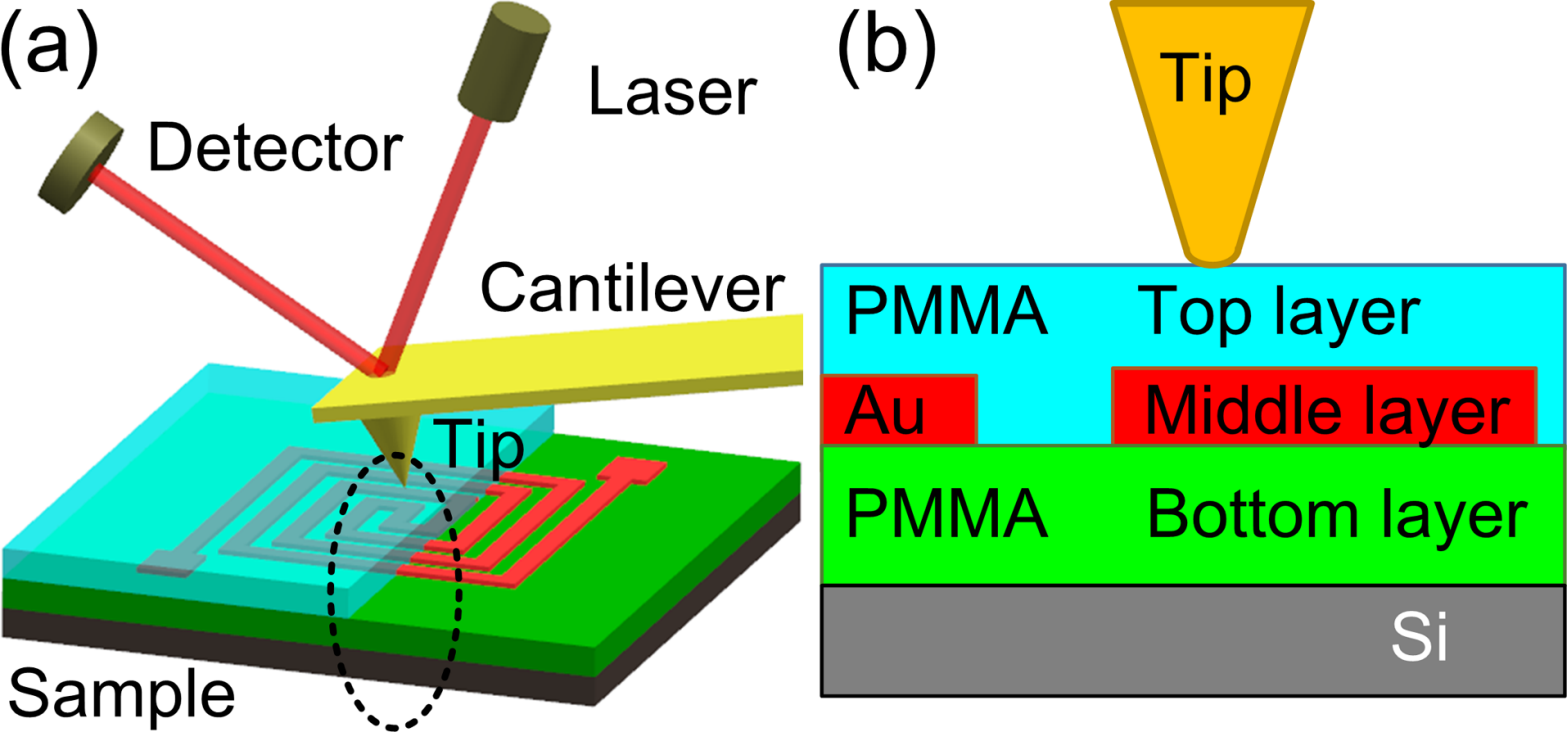
(a) Schematic illustration of CR-AFM imaging on the flexible circuit sample. (b) An enlarged sectional view of the contact between the AFM tip and the multilayer structure.

We fabricated a set of samples having a PMMA–Au–PMMA sandwich structure with a thick PMMA film as the bottom layer, a patterned Au layer as the middle, functional circuit layer, and a thin PMMA film as the top protection layer, as schematically shown in Figure 1. The bottom layer was first coated on a clean silicon substrate by spin casting 11 wt % PMMA in anisole solvent at 1500 rpm for 5 min, resulting in a thickness of approximately 3.5 µm. Then, a 300 nm thick Au film was sputtered on the PMMA substrate by using magnetron sputtering. The Au film was subsequently patterned by focused ion beam (FIB) milling (FEI, Helios NanoLab 650). In FIB processing, the ion acceleration voltage and the beam current were 30 kV and 2.5 nA, respectively. The effective area of the circuit pattern was 55 × 55 µm. Finally, samples having different top layer thicknesses were obtained by spinning a more diluted PMMA solution (2–6%) with a speed ranging from 1000 to 6000 rpm. The resulting cover thicknesses were 52, 117, 185, 380, and 653 nm as measured by AFM. Furthermore, the top surface was smoothed to eliminate the cross-talk of surface topography in subsurface CR-AFM imaging.

Our experiments were performed on an MFP-3D Origin AFM (Asylum Research, Santa Barbara, CA) with the dual AC resonance tracking (DART) module applied for CR-AFM subsurface imaging. Three types of cantilevers were used: ContAl-G (BudgetSensors, Innovative Solutions Bulgaria Ltd., Bulgaria), PPP-FM (NanoSensors, NanoWorld AG, Switzerland) and ASYELEC-01 (Oxford Instruments, Asylum Research, Santa Barbara, CA). Their corresponding parameters are presented in Table 1, where *L*, *L*_2_, *h*, *k*_C_, *f*_1_^0^ and *f*_2_^0^ are the cantilever length, the length from the tip position to the free end, the tip height, the cantilever stiffness and the free resonance frequencies of the first and the second eigenmodes, respectively. The cantilever stiffness and the free resonance frequency were calibrated by utilizing the thermal noise method while others were provided by the respective manufacturers.

**Table 1:** Parameters of the experimental cantilevers.

	*L* (µm)	*L*_2_ (µm)	*h* (µm)	*k*_C_ (N/m)	*f*_1_^0^ (Hz)	*f*_2_^0^ (Hz)

ContAl-G	450	15	17.0	0.30	15414	97595
PPP-FM	225	15	12.5	0.84	52127	319948
ASYELEC-01	240	16	14.0	2.58	76106	492502

### Multilayer contact model

The axisymmetric indentation of a tip contacting with a multilayered elastic half-space sample as illustrated in Figure 1b is too complicated for a simple analytical solution. Many efforts have been tried to solve this kind of contact problem. The contact stiffness and the equivalent indentation modulus are usually calculated via approximated approaches [29–30], empirical/fitted formulas [31–33], and semi-analytical solutions [34–36]. These methods are constrained to cases such as a two-layer system [32,37], or an infinite rigid substrate [30,38]. However, the semi-analytical solutions proposed by Constantinescu et al. [34] and Stan et al. [36] allow convenient modeling of the contact mechanics for multilayers without the requirement of a rigid substrate. In their approach, the stress and displacement fields for each layer and the substrate are first expressed in terms of harmonic Papkovich–Neuber potentials. Then, the unknown functions in the potential formulas are determined from the boundary conditions at the surface and at the interface between adjacent layers. Perfect bonding and free sliding are assumed for the layer-to-layer contact, which transmit displacement and stress from one layer to the next. Considering a frictionless contact between the rigid tip and the first layer and combining with the interface boundary conditions, the principal unknown function is acquired as the solution of a Fredholm integral equation of the second kind. Finally, the contact radius and the apparent contact modulus are calculated based on the assumption that the surface traction vanishes at the edge of contact zone. Here, the apparent contact modulus *M*_app_ is defined as the modulus supposing that the tip is infinitely rigid. More detailed calculation procedures can be found in the references [34,36]. A similar, quantitative analysis method has also been employed by Stan et al. [39].

The circuit pattern in our experiments has a width of 2 µm, which is far larger than the contact radius. Therefore, the sample can be regarded as an elastic multilayer film with an infinite lateral width. The method proposed by Constantinescu et al. [34] is thus utilized to calculate the contact stiffness. Because the probe tip has a modulus that is not infinite, we modified the apparent contact stiffness *M*_app_ to the effective contact modulus *M*_eff_ as,

[1] $$M_{\text{eff}}=1/M_{\text{app}}+\left(1-v_{\text{tip}}^{2}\right)/E_{\text{tip}},$$

where *E*_tip_ and *v*_tip_ are the Young’s modulus and the Poisson’s ratio of the AFM tip, respectively. The normal contact stiffness, *k*_N_, is evaluated as follows for a blunt conical tip [35],

[2] $$k_{\text{N}}={\left[\left(8M_{\text{eff}}F_{\text{N}}\right)/\left(\pi \cot\alpha \right)\right]}^{1/2},$$

where *F*_N_ is the normal force and α is the half-cone angle of the tip. It should be mentioned that Equation 2 was originally used for a sharp conical tip. In calculating the indentation depth and the contact radius, the tip radius is also taken into account for a blunt conical tip [34–35].

In our calculations, a half-cone angle of 15° was determined for the adopted probes, which was in accordance with the manufacturer’s specifications. The tip apex radius was averaged to be approximately 50 nm according to scanning electron microscopy measurements on some of the probes after CR-AFM subsurface imaging experiments. The thicknesses of the bottom, middle and top layers were approximated from practical AFM measurements. Different layer materials were investigated including PMMA, high-density polyethylene (HDPE), polystyrene (PS), polycarbonate (PC) and polyimide (PI) for the bottom and top layers, and Au, Cu, Ti, Ag and Mg for the middle layer. The mechanical parameters of these materials are listed in Table 2. The Young’s modulus and Poisson’s ratio of the silicon substrate are 160 GPa and 0.278, respectively.

**Table 2:** Mechanical parameters of different materials used in the theoretical analysis and FEA calculations.

	Au	Cu	Ti	Ag	Mg	PMMA	HDPE	PS	PC	PI

*E*/GPa	74	130	116	83	45	6.5	0.85	3.3	2.2	2.5
*v*	0.42	0.34	0.32	0.37	0.29	0.39	0.46	0.35	0.37	0.34

### Cantilever vibration model

The experimental contact stiffness was determined from the tracked contact resonance frequency according to the well-known Euler–Bernoulli cantilever vibration model [40–41]. In this model, one end of the cantilever is clamped while the other end with the tip is in contact with the sample. The tip position, the cantilever tilt caused by mounting and the effect of lateral forces are all considered [14–16]. The elastic interactions between the tip and the sample are modeled with contact stiffnesses in the normal and lateral directions. The damping is neglected because it does not have significant influence on the resonance frequency. The contact stiffness is determined by solving the equation,

[3] $$3A\left(k_{\text{L}}/k_{\text{N}}\right)\left(k_{\text{N}}/k_{\text{C}}\right)+B_{1}+B_{2}\left(k_{\text{L}}/k_{\text{N}}\right)+\left(C/3\right)\left(k_{\text{C}}/k_{\text{N}}\right)=0,$$

where

[4] $$A={\left(h/L_{\text{1}}\right)}^{2}\left(1-\cos\lambda_{\text{n}}L_{1}\cosh\lambda_{\text{n}}L_{1}\right)\left(1+\cos\lambda_{\text{n}}L_{2}\cosh\lambda_{\text{n}}L_{2}\right)$$

[5] $$B_{1}=\sin^{2}\alpha_{0}B_{1}^{*}-B_{2}^{*}+\cos^{2}\alpha_{0}B_{3}^{*}$$

[6] $$B_{2}=\cos^{2}\alpha_{0}B_{1}^{*}+B_{2}^{*}+\sin^{2}\alpha_{0}B_{3}^{*}$$

[7] $$C=2{\left(\lambda_{\text{n}}L_{1}\right)}^{4}\left(1+\cos\lambda_{\text{n}}L\cosh\lambda_{\text{n}}L\right)$$

with

[8] $$B_{1}^{*}={\left(h/L_{1}\right)}^{2}{\left(\lambda_{\text{n}}L_{1}\right)}^{3}\left[\begin{array}{l} \left(1+\cos\lambda_{\text{n}}L_{2}\cosh\lambda_{\text{n}}L_{2}\right)\\ \left(\begin{array}{l} \sin\lambda_{\text{n}}L_{1}\cosh\lambda_{\text{n}}L_{1}\\ +\cos\lambda_{\text{n}}L_{1}\sinh\lambda_{\text{n}}L_{1} \end{array}\right)-\\ \left(1-\cos\lambda_{\text{n}}L_{1}\cosh\lambda_{\text{n}}L_{1}\right)\\ \left(\begin{array}{l} \sin\lambda_{\text{n}}L_{2}\cosh\lambda_{\text{n}}L_{2}\\ +\cos\lambda_{\text{n}}L_{2}\sinh\lambda_{\text{n}}L_{2} \end{array}\right) \end{array}\right]$$

[9] $$\begin{array}{c} B_{2}^{*}=\left(h/L_{1}\right){\left(\lambda_{\text{n}}L_{1}\right)}^{2}\sin\alpha_{0}\cos\alpha_{0}\\ \left[\begin{array}{l} \left(1+\cos\lambda_{\text{n}}L_{2}\cosh\lambda_{\text{n}}L_{2}\right)\sin\lambda_{\text{n}}L_{1}\sinh\lambda_{\text{n}}L_{1}+\\ \left(1-\cos\lambda_{\text{n}}L_{1}\cosh\lambda_{\text{n}}L_{1}\right)\sin\lambda_{\text{n}}L_{2}\sinh\lambda_{\text{n}}L_{2} \end{array}\right] \end{array}$$

[10] $$B_{3}^{*}=\lambda_{\text{n}}L_{1}\left[\begin{array}{l} \left(1+\cos\lambda_{\text{n}}L_{2}\cosh\lambda_{\text{n}}L_{2}\right)\\ \left(\sin\lambda_{\text{n}}L_{1}\cosh\lambda_{\text{n}}L_{1}-\cos\lambda_{\text{n}}L_{1}\sinh\lambda_{\text{n}}L_{1}\right)-\\ \left(1-\cos\lambda_{\text{n}}L_{1}\cosh\lambda_{\text{n}}L_{1}\right)\\ \left(\sin\lambda_{\text{n}}L_{2}\cosh\lambda_{\text{n}}L_{2}-\cos\lambda_{\text{n}}L_{2}\sinh\lambda_{\text{n}}L_{2}\right) \end{array}\right]$$

Here, *L*_1_ = *L* – *L*_2_ and λ_n_*L* is the normalized wave number of the *n*th flexural resonance, which can be calculated from the contact resonance frequency *f*_n_^c^ and the free resonance frequency *f*_n_^0^ with the relation,

[11] $${\left(\lambda_{\text{n}}L\right)}^{\text{c}}={\left(\lambda_{\text{n}}L\right)}^{\text{0}}\sqrt{f_{\text{n}}^{\text{c}}/f_{\text{n}}^{\text{0}}}.$$

In our analysis, a cantilever tilt angle, α_0_, of 11° was used. The ratio of the lateral contact stiffness to the normal one *k*_L_/*k*_N_ was set to 0.86. Other parameters of the cantilever and the tip can be found in Table 1.

Since different cantilevers and vibration eigenmodes are employed for subsurface imaging, direct comparisons of the measured resonance frequency contrasts are not suitable. Therefore, we would like to use the contact stiffness contrast to evaluate the imaging quality. A normalized contrast metric is adopted as follows [27],

[12] $$c=\left(k_{\text{ckt}}-k_{\text{sub}}\right)/k_{\text{sub}}\times 100\%\;,$$

where *k*_ckt_ and *k*_sub_ are the evaluated contact stiffnesses when the tip is positioned right above the circuit layer and on the polymer medium, respectively.

In the followed analysis, a tip radius of 50 nm and a half-cone angle of 15° are used unless otherwise noted. In fact, the tip radius may vary, which affects the quantitative CR-AFM measurements [42]. We have calculated the contact stiffness contrast when the tip radius ranged from 6 to 121 nm. Such a range covers the practical tip radius in experiments according to scanning electron microscopy imaging. The results show that deviations in the evaluated contact stiffness contrast are less than 1% in this radius range even when a large normal force of 1000 nN is applied for the typical sample with a cover layer thickness of 50 nm. Therefore, we can safely assume the tip radius of 50 nm without significant errors. Similar calculations also demonstrated that the approximation of a sharp conical tip to a blunt conical tip does not lead to large deviations in stiffness contrast evaluation, especially under a large indentation depth.

### Finite element analysis

The FEA method was additionally used to simulate the contact between the tip and the multilayer flexible circuit. The structural model was the same as that in the analytical calculations. Since the model is axisymmetric, we used the two-dimensional FEA simulations for simplicity. The width of the sample was set to 2 µm and the thickness of the silicon substrate was 10 µm. Mesh refinement was applied near the tip–sample contact area. The FEA computations were performed by using the commercial Ansys software. The PLANE183-type element was utilized to model the tip and sample. The TARGE169 and the CONTA172 elements were adopted to handle the surface-to-surface contact. The adjacent layers were perfectly bonded. Contact force was applied on the tip and the bottom of the Si substrate was fixed. Because the contact stiffness is the derivative of loading force to elastic deformation, we used the following approximation [43],

[13] $$k\left(F_{i}\right)=\partial F_{i}/\partial d_{i}≅\left(\begin{array}{l} \Delta F_{i-1}\Delta d_{i}/\Delta d_{i-1}\\ +\Delta F_{i}\Delta d_{i-1}/\Delta d_{i} \end{array}\right)/\left(\Delta d_{i}+\Delta d_{i-1}\right)\;.$$

Here, Δ*F_i_* is the difference of the applied forces between load steps *i* + 1 and *i*, and Δ*d_i_* is the difference of the corresponding sample deformations at the position of the tip apex. The contact stiffness on the polymer substrate was solved by utilizing the same model, but with the middle layer material changed to PMMA. The mechanical properties of the materials in FEA calculations can be found in Table 2.

## Results and Discussion

### Subsurface imaging

**Figure 2:**
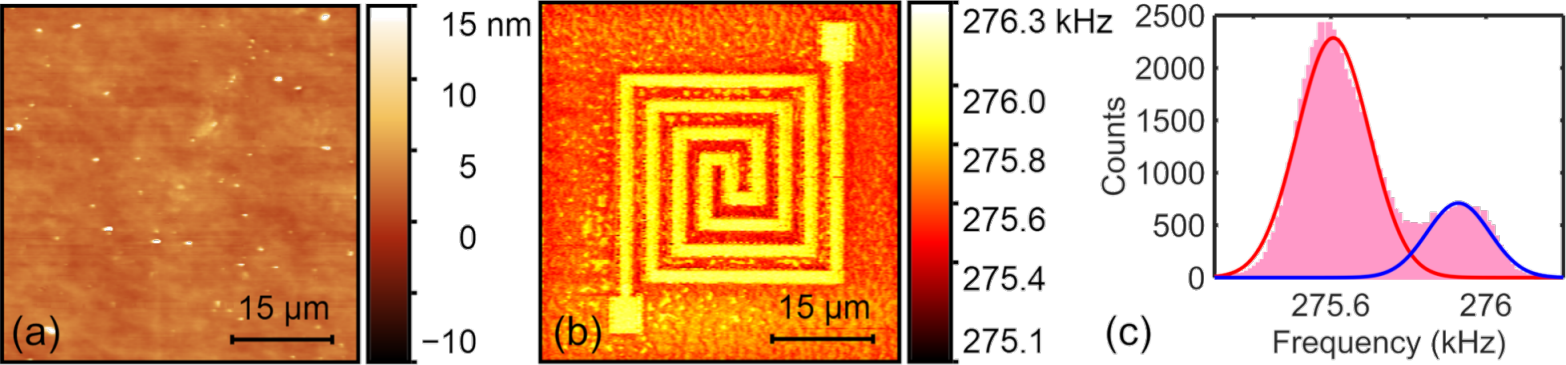
Subsurface imaging of the flexible circuit sample with a top layer thickness of 52 nm measured via the CR-AFM method. (a) Topography. (b) The first contact resonance frequency. (c) Histogram of the resonance frequency image. The results were obtained by using the PPP-FM probe under a normal force of 74 nN.

The flexible circuit sample with a 52 nm thick top layer was first imaged by CR-AFM to ascertain its subsurface imaging capability. The PPP-FM probe was used with an applied normal force of approximately 74 nN and the first contact resonance frequency was tracked. The obtained topography and resonance frequency images are shown in Figure 2a and Figure 2b, respectively. We can clearly discern the circuit pattern from the resonance frequency image, which cannot be found in the topography. This verifies the CR-AFM’s capability in detecting subsurface circuit structures. In Figure 2c, we show the histogram of the resonance frequency image (Figure 2b), which demonstrates two peaks at 275.60 ± 0.10 and 275.92 ± 0.08 kHz. They correspond to the measured resonance frequency on the polymer matrix and above the embedded Au layer, respectively. The resonance frequency shift can be unambiguously distinguished. The contact stiffnesses were evaluated to be 151.4 ± 0.5 N/m above the circuit and 149.6 ± 0.5 N/m on the polymer substrate, resulting in a contrast metric of 1.20 ± 0.67% according to Equation 12.

### Influence of experimental parameters

In order to optimize the imaging setup for better subsurface contrast, we investigated the effects of some major experimental parameters, including the normal force, cantilever stiffness and vibration eigenmode.

#### Normal force

**Figure 3:**
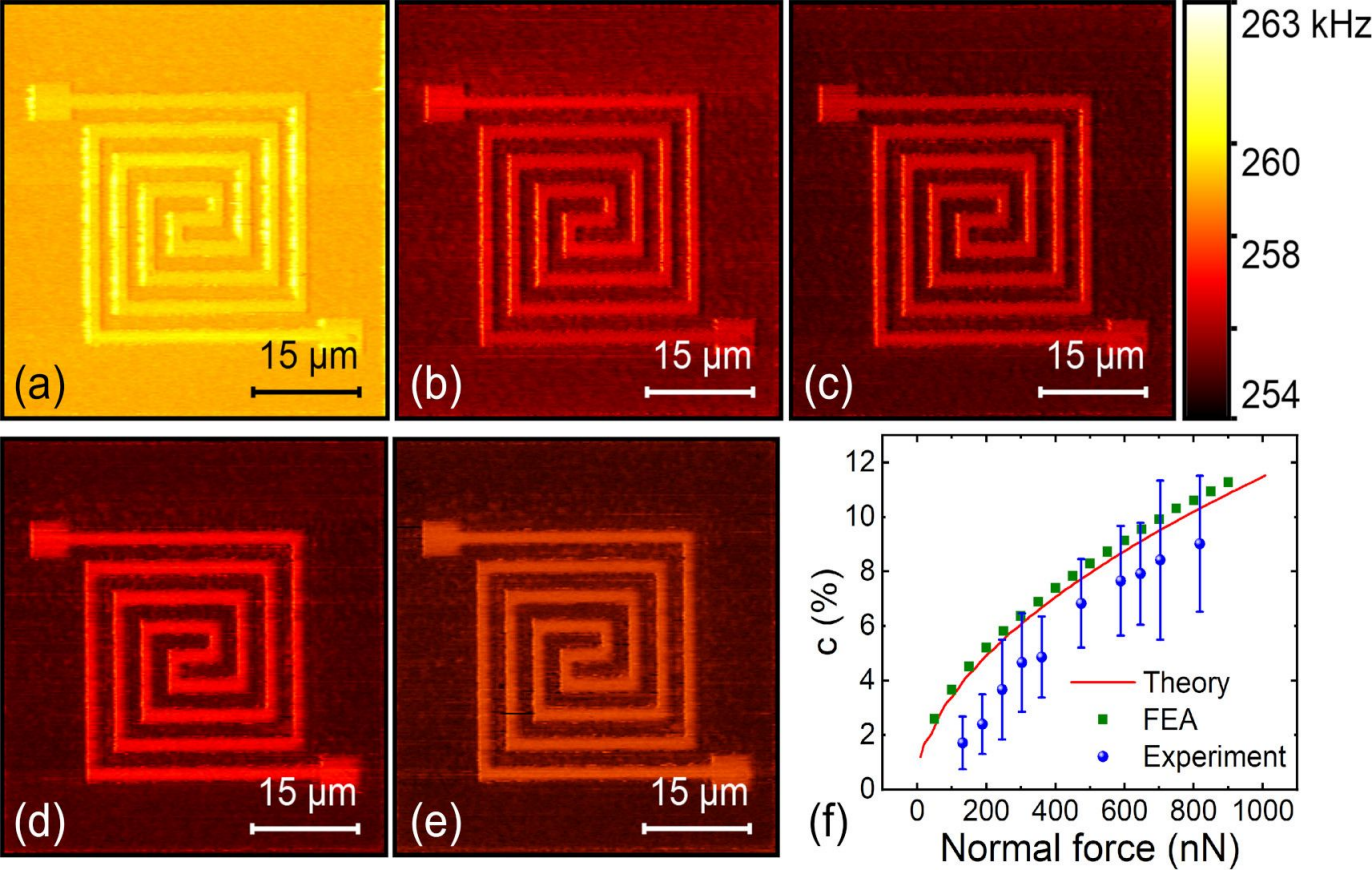
Influence of the applied force on CR-AFM subsurface imaging of the flexible circuit sample with a 52 nm thick top layer. (a–e) The first contact resonance frequency images at the applied force of 132, 303, 360, 589, and 646 nN, respectively, using the PPP-FM cantilever. (f) The obtained contact stiffness contrast as a function of normal force from the experiments, theoretical calculations and FEA simulations.

We first performed subsurface imaging on the sample with a 52 nm thick top layer under different normal forces. The PPP-FM cantilever was adopted and the normal forces were applied in a range from 132 to 818 nN. Figure 3a–e exhibits the first contact resonance frequency images under the normal forces of 132, 303, 360, 589 and 646 nN, respectively. Intuitively, a larger imaging contrast is observed with increasing normal force. Among the different subsurface images, a slight decrease in the resonance frequency appears occasionally, which is in contradiction with the general knowledge that a larger force induces a larger contact resonance frequency. Such a phenomenon may be induced by the errors in resonance frequency tracking by using the DART mode, especially when certain oscillation nonlinearity, spurious frequency peaks and large noise are presented in the contact resonance spectra. However, the frequency decrease with increasing normal force is generally not so significant, and the contrast between areas with and without subsurface structure in the same image remain reliable. More quantitatively, the evaluated stiffness contrasts together with the results of the theoretical and FEA calculations are shown in Figure 3f. The experimental, theoretical and FEA results agree well with each other on the general trends. All of them demonstrate that a larger force induces a larger stiffness contrast and the rate of contrast increase slows down along with the force increment. The deviations between the experimental results and the theoretical calculations are suggested to be caused by the simplified assumptions in the models and the errors of model parameters, including tip shape, mechanical parameters and boundary conditions. The theoretical and FEA computations are in close accordance, especially in the small force range. Discrepancies gradually appear with further increase in the normal force, which is possibly induced by the approximations in the theoretical calculations.

#### Cantilever stiffness

**Figure 4:**
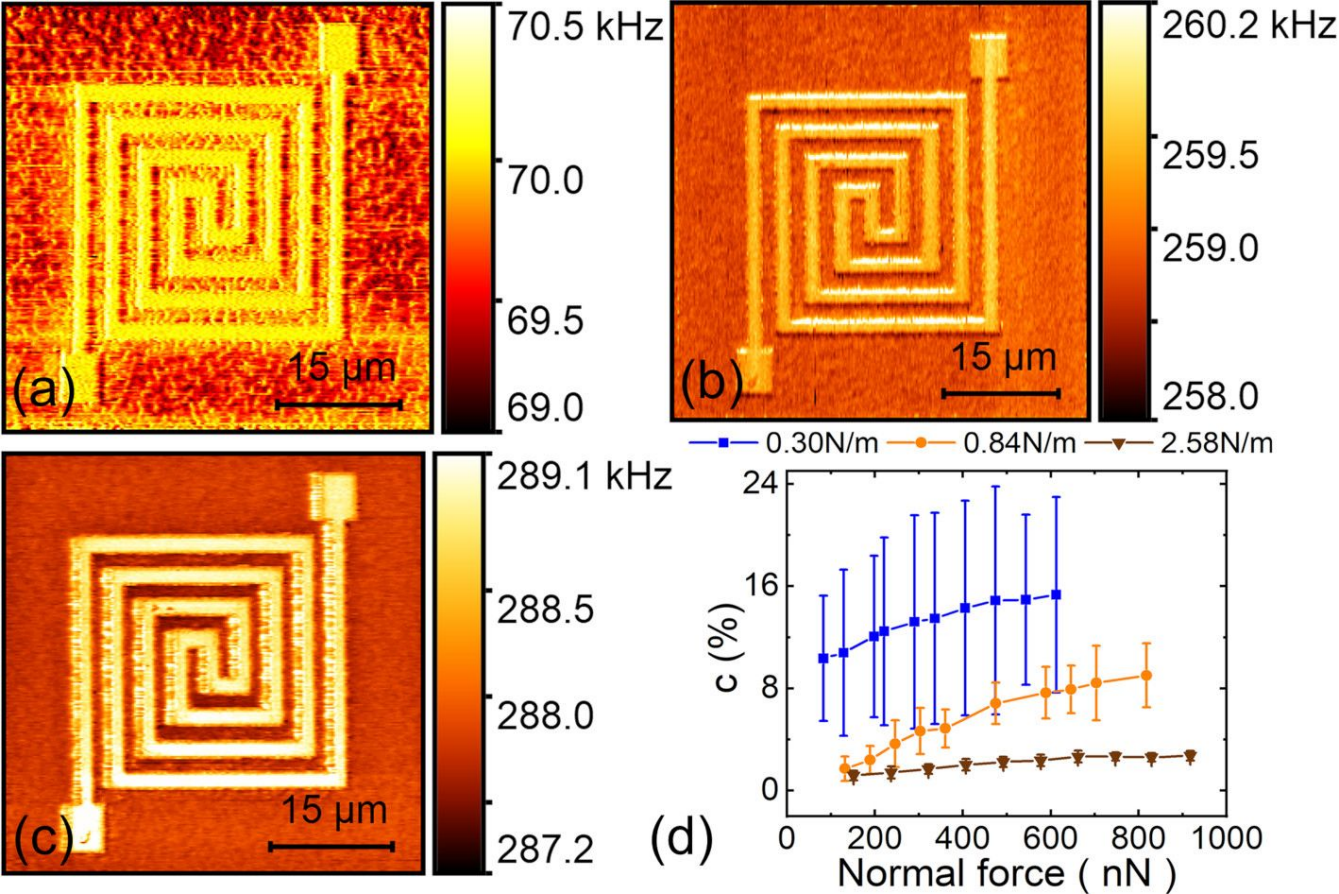
Influence of cantilever stiffness on CR-AFM subsurface imaging of the flexible circuit sample with a 52 nm thick top layer. (a–c) The first contact resonance frequency images of three cantilevers having stiffness of 0.30, 0.84 and 2.58 N/m, respectively. The applied normal forces were approximately 221, 189 and 237 nN. (d) Evaluated contact stiffness contrasts for the three probes under various normal forces.

The frequency sensitivity of contact resonance to local mechanical properties also depends on the cantilever stiffness. We used three types of cantilevers for subsurface imaging whose spring constants were 0.30, 0.84 and 2.58 N/m, respectively. Other parameters are listed in Table 1. Figure 4a–c shows the resonance frequency images of using the three cantilevers under close normal forces of approximately 221, 189 and 237 nN. The experiments were performed on the flexible circuit sample with a 52 nm thick top layer. A quantitative comparison of the contact stiffness contrast is presented in Figure 4d. We can find that the contrast acquired by the cantilever with the stiffness of 0.30 N/m is the largest, while the one with the stiffness of 2.58 N/m is the smallest. This result indicates that using a softer cantilever is generally better to achieve a higher subsurface contrast for the flexible circuit sample under nearly the same experimental conditions. Although the contrast with a softer cantilever is much better, the imaging noise is also higher as shown in Figure 4d. In practice, choosing an optimal cantilever stiffness can be achieved through analysing the cantilever dynamics, which is able to establish the quantitative dependence of the resonance frequency sensitivity on the contact stiffness.

#### Cantilever oscillation eigenmode

**Figure 5:**
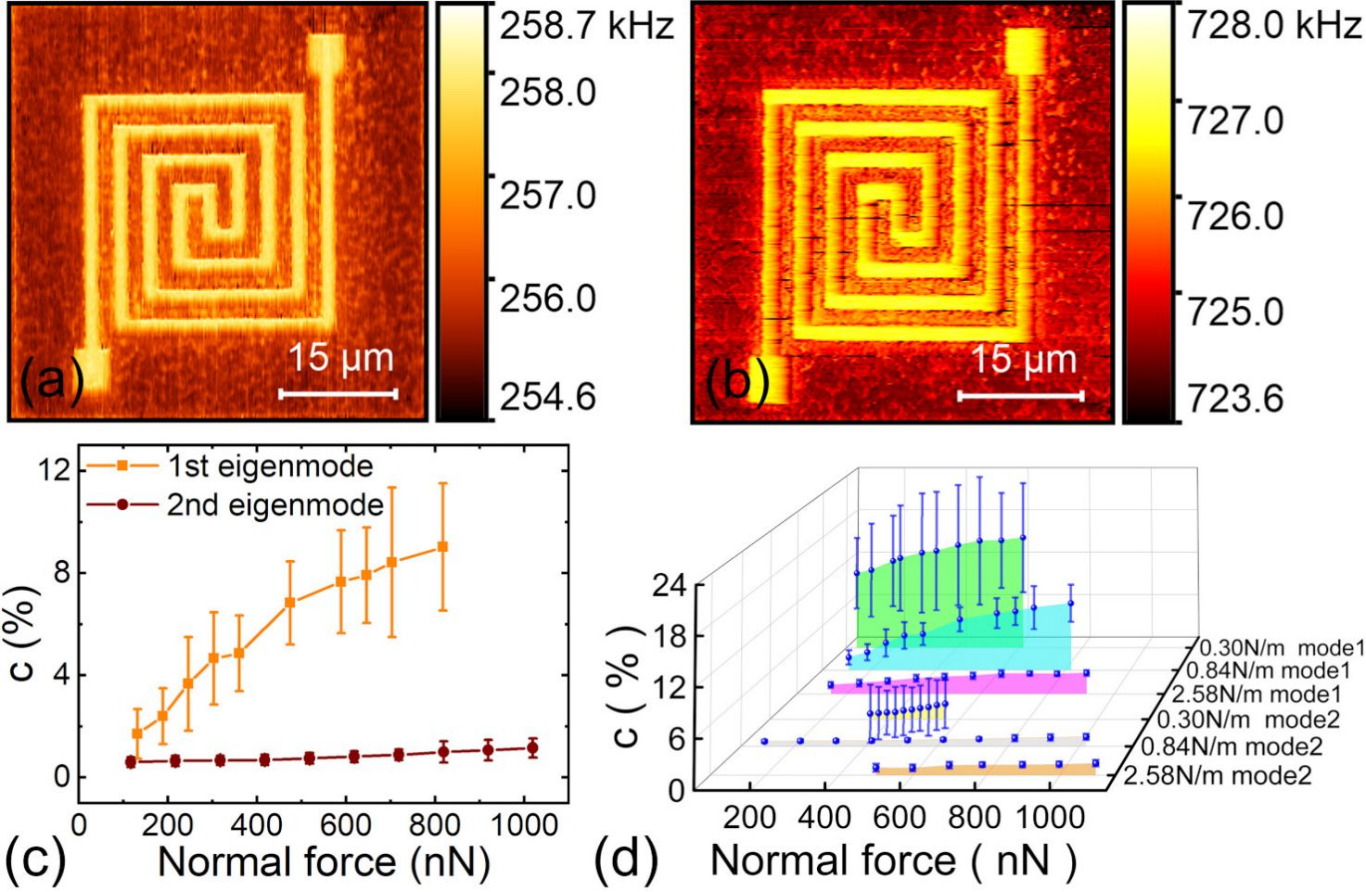
The influence of cantilever oscillation eigenmode on CR-AFM subsurface imaging of a flexible circuit sample with a top layer thickness of 52 nm. (a,b) Tracked resonance frequency images of the first and the second eigenmodes of the PPP-FM probe under a normal force of 818 nN. (c) Experimental contact stiffness contrast of the first two eigenmodes under various normal forces. (d) Stiffness contrast when using different cantilevers and different eigenmodes.

The employed cantilever oscillation mode is another important factor in CR-AFM imaging. We applied the first two eigenmodes of the PPP-FM cantilever to scan the flexible circuit sample with a top layer thickness of 52 nm. The results are shown in Figure 5a,b with the same normal force of approximately 818 nN. A more quantitative comparison of the respective contact stiffness contrast is demonstrated in Figure 5c where different normal forces have been used. From all the experiments, much better stiffness contrasts and larger uncertainties can be found for the first eigenmode. Owing to the relatively soft materials employed in the flexible circuits, a softer cantilever stiffness gives better sensitivity as can be seen from previous discussions for the cantilever stiffness effects. Vibrating at the second mode has a significantly larger effective spring constant than the fundamental one. This leads to reduced contrast. In Figure 5d, all the experimental results of different eigenmodes and cantilevers are summarized. It is clear that the first eigenmode of a softer cantilever can improve the detected contact stiffness contrast and thus benefit subsurface imaging of the flexible circuits. On the contrary, a larger effective probe stiffness using either a stiffer cantilever or the second eigenmode produces a small stiffness contrast.

### Influence of sample properties

In addition to the experimental parameters, we also investigated the influence of different sample properties on subsurface imaging. Factors including the material parameters and the thickness of each layer were mainly considered.

#### Cover film thickness

**Figure 6:**
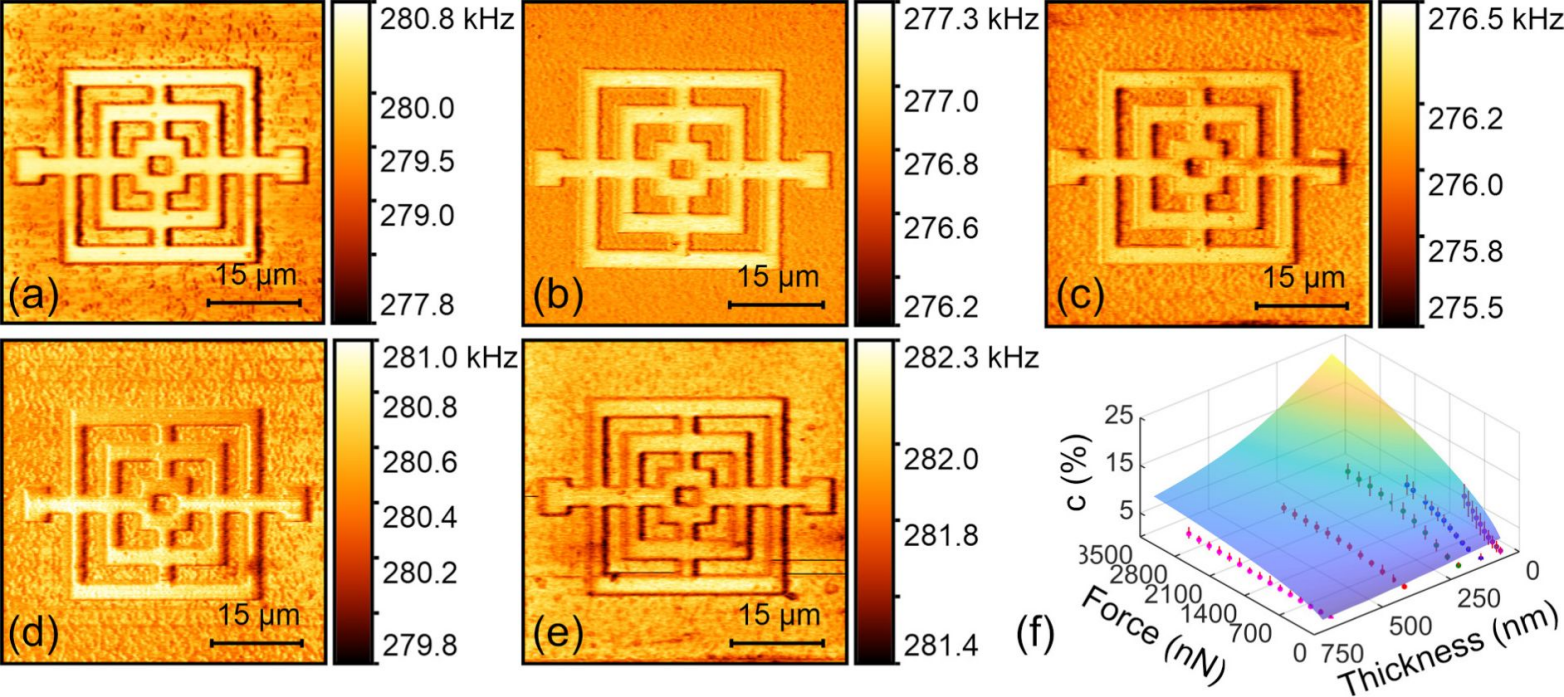
Influence of cover thickness on CR-AFM subsurface imaging of the circuit sample. (a–e) Frequency images of samples with the cover layer thicknesses of 52, 117, 185, 380 and 653 nm, respectively. The images were acquired by using the first eigenmode of the PPP-FM cantilever. The applied forces were 415, 376, 427, 441 and 400 nN, respectively. (f) Contact stiffness contrasts under different cover thicknesses and applied forces. The scatter plots denote the experimental results and the mesh surface is constructed from theoretical calculations.

CR-AFM imaging of subsurface flexible circuits was carried out on samples with top layer thicknesses of 52, 117, 185, 380 and 653 nm, respectively. The acquired frequency images are shown in Figure 6a–e. Here, the first eigenmode of the PPP-FM cantilever was used with similar applied forces of 415, 376, 427, 441 and 400 nN, respectively. For the thinnest top layer, the buried Au layer can be clearly distinguished from the background, as shown in Figure 6a. However, with the increase of the cover thickness to 653 nm, the circuit pattern fades out gradually, indicating a decreased contact stiffness contrast. In Figure 6f, we show the experimental and theoretical contact stiffness contrasts for the buried Au layers under different cover thicknesses and loading forces. In general, a larger force and a lower cover thickness results in a higher stiffness contrast. The experimental contrasts are lower than the theoretical ones, yet their general trends agree considerably well. Moreover, for a specified cover film thickness, the contact stiffness contrast plateaus when applying large loading forces. Occasionally, a larger frequency is observed for a thicker cover layer under similar experimental conditions. This may be induced by the resonance frequency tracking errors in DART operation mode and also the thermal drift.

#### Other sample properties

**Figure 7:**
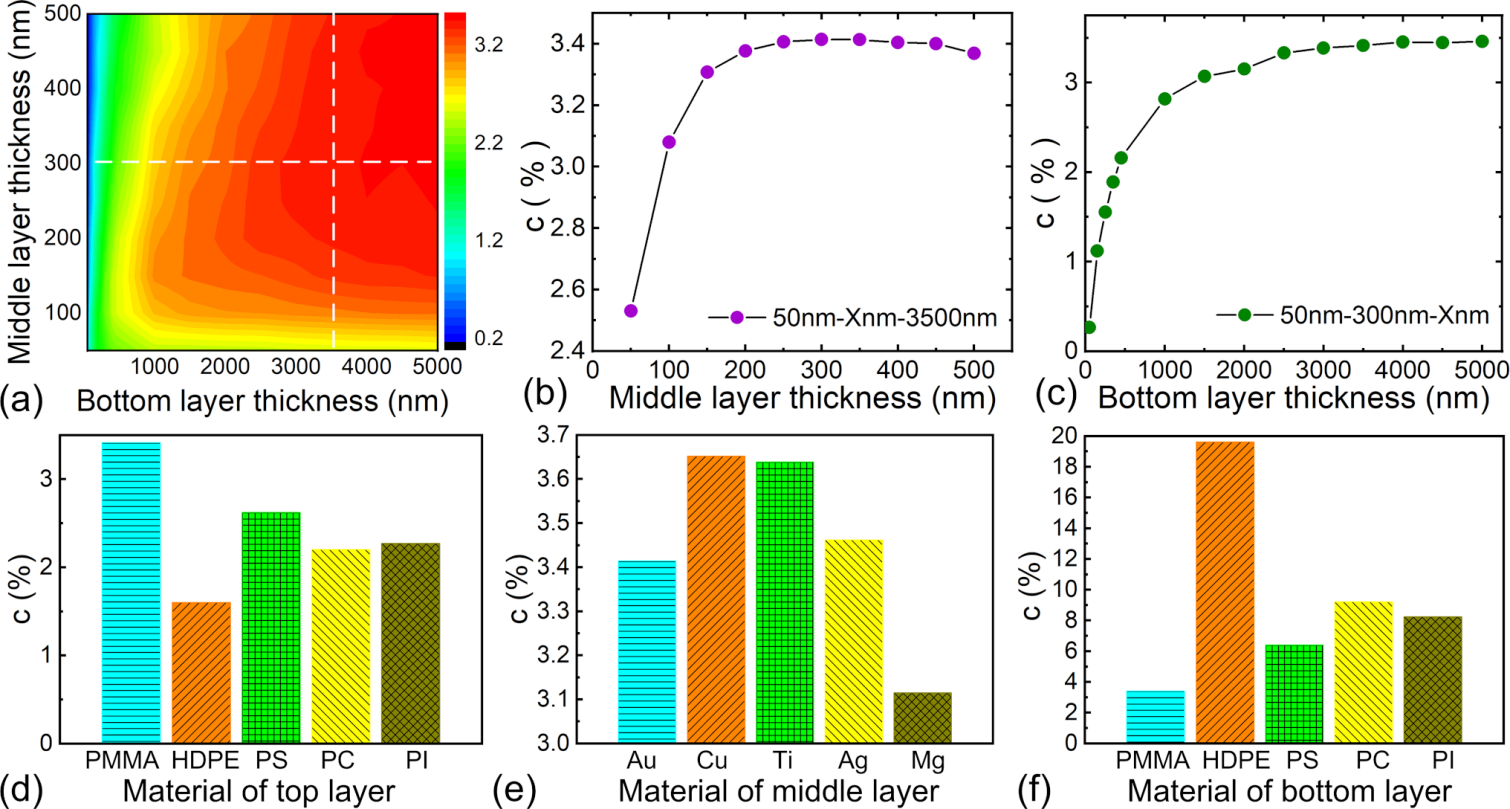
Influence of thickness and material properties of each layer on subsurface imaging of the circuit pattern. (a) Theoretical stiffness contrasts for different middle layer and bottom layer thicknesses. The theoretical calculations were made on the PMMA–Au–PMMA structures with a top layer thickness of 50 nm under a normal force of 100 nN. (b,c) Cross-section profiles at the middle layer thickness of 300 nm and the bottom layer thickness of 3500 nm, respectively. (d–f) Calculated contact stiffness contrasts when different materials are used for the top, middle and bottom layers. The thicknesses are 50, 300 and 3500 nm for the top, middle and bottom layers, respectively.

In addition to the cover layer thickness, the influence of other sample properties, including the material properties and the thickness of each layer, was investigated via systematic theoretical analysis. To do this, a blunt conical tip with a half-cone angle of 15° and a tip radius of 50 nm was used to indent the multilayered sample. First, the effects of the middle and bottom layer thicknesses were studied on the PMMA–Au–PMMA structures by setting them as independent variables while calculating the contact stiffness contrast. Here, a cover layer thickness of 50 nm and a normal force of 100 nN were selected. The resulting contact stiffness contrast is shown in Figure 7a as a function of the middle layer and bottom layer thicknesses. Cross-section profiles of Figure 7a at a middle layer thickness of 300 nm and a bottom layer thickness of 3500 nm are presented in Figure 7b and Figure 7c, respectively. It is obvious that a thicker bottom layer induces a better imaging contrast and the contrast plateaus at a higher thickness. Such a behaviour is due to the influence of the Si substrate. When the bottom layer becomes thicker, the substrate effect is less significant. For the middle layer thickness, there is an inflection point at approximately 300 nm. The contact stiffness contrast first drastically increases with increasing middle layer thickness and then decreases slightly after the inflection point is reached, which is also relevant with the substrate effect.

Second, the influence of the mechanical properties of each layer was additionally investigated. Polymers including PMMA, HDPE, PS, PC and PI were used for the top and bottom layers, while the metallic materials Au, Cu, Ti, Ag and Mg were used for the circuits. The Young’s modulus and Poisson’s ratio of the considered materials are listed in Table 2. The thicknesses were 50, 300 and 3500 nm for the top, middle and bottom layers, respectively. The calculated contact stiffness contrasts are shown in Figure 7d–f. Among these materials, copper is frequently used to fabricate flexible circuits and PI is often serves as the substrate material in industrial productions. From the results, it can be concluded that for the layer thicknesses in our experiments, a stiffer cover layer and a softer bottom layer can enable much easier subsurface sensing of the flexible circuits via CR-AFM, and the middle layer material does not have much influence.

#### Optimization of CR-AFM subsurface imaging

**Figure 8:**
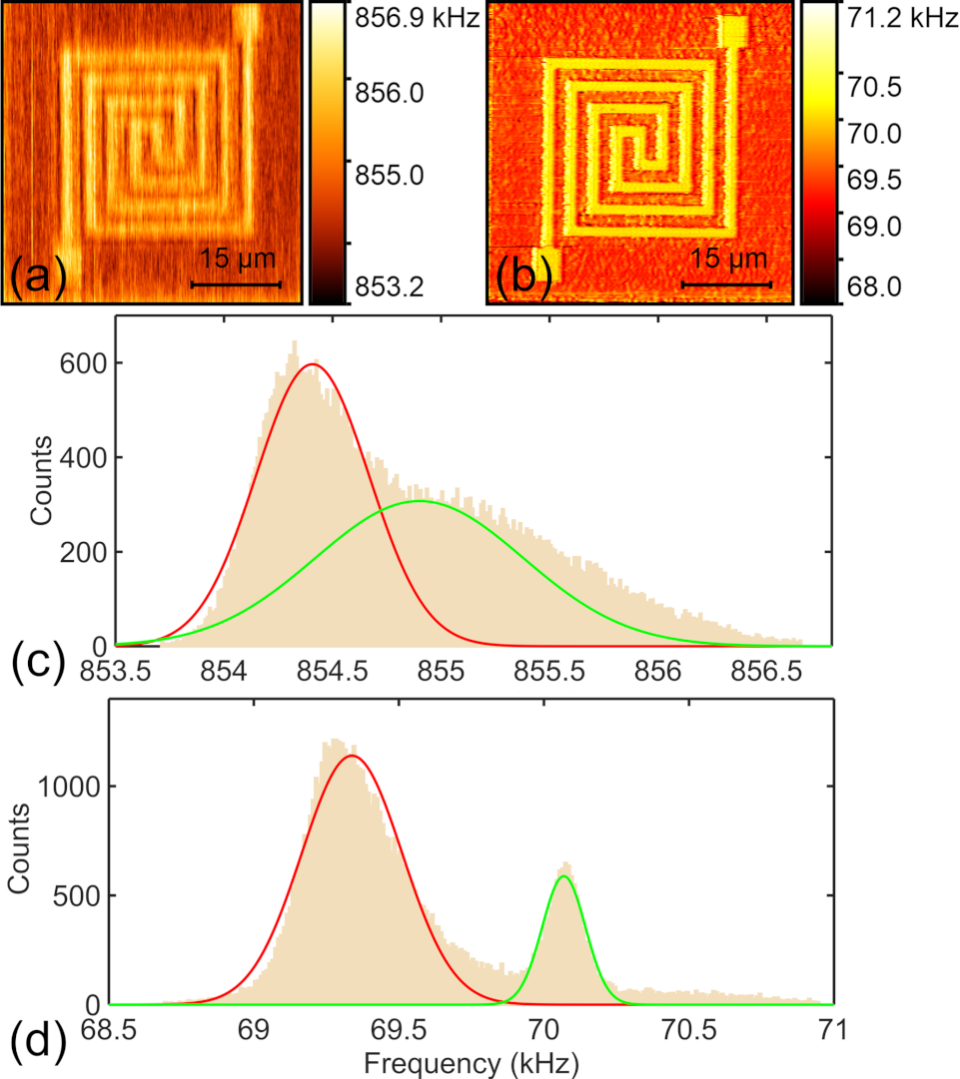
Optimization of CR-AFM subsurface imaging on a flexible circuit sample. (a,b) CR-AFM frequency images obtained before and after optimization, respectively. (c,d) Corresponding histograms of the images shown in (a) and (b). All the measurements were performed on the flexible circuit sample with a top layer thickness of 78 nm. For (a), the second mode of an ASYELEC-01 cantilever was used with an applied normal force of 398 nN. The image in (b) was obtained with the first mode of a ContAl-G probe under a tip-generated load of 613 nN.

Based on the systematic investigation of the main variables, a contrast optimization for subsurface imaging of the circuit patterns is expected. Typical demonstrations are presented in Figure 8a and Figure 8b, which are CR-AFM frequency images for a specific flexible circuit sample before and after optimization, respectively. The corresponding frequency histograms are demonstrated in Figure 8c and Figure 8d. The experiments were carried out on the sample with a top layer thickness of 78 nm. Figure 8a was acquired by using the second eigenmode of an ASYELEC-01 cantilever, which has a stiffness of 2.58 N/m. A normal force of 398 nN was applied in scanning. These imaging parameter settings were somewhat arbitrarily selected. It can be found that the embedded Au pattern is observable but with a quite low contrast. According to the previous investigations, a softer cantilever and a lower eigenmode could enhance the detected stiffness contrast. Therefore, we changed to the softer ContAl-G cantilever with the stiffness of approximately 0.30 N/m and employed its first mode. In addition, the normal force was increased to 613 nN. A typical CR-AFM frequency image is shown in Figure 8b. A significant contrast improvement is observed for the subsurface circuit pattern. This can be clearly seen from the histograms in Figure 8c,d. Quantitative evaluations show that the stiffness contrast increases from 0.2 ± 0.2% to 13.8 ± 5.0% after optimization. Generally, the optimization can be achieved by setting the variables so that the system provides the best sensitivity according to the dispersion curve. The cantilever dynamics analysis and the tip–sample contact mechanics model can provide the dispersion relation between contact resonance frequency and contact stiffness, and the relationship between contact stiffness and local mechanical properties, respectively [16,44]. The former one provides the optimization guide on parameter selections and the latter one relates the specifications of the sample properties [34–36]. The theoretical analyses together with the systematic experimental results demonstrated in this work can provide a general guide for proper selection of the experimental settings.

#### Defect detection

**Figure 9:**
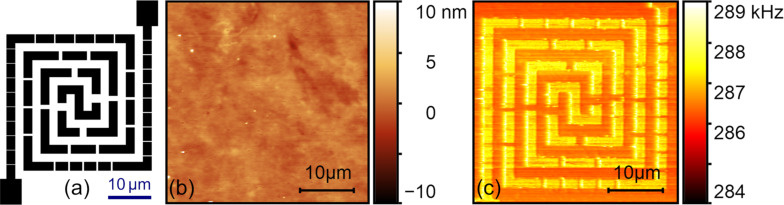
Subsurface imaging of defects in the buried circuit pattern using CR-AFM. (a) Schematic illustration of the introduced defects in the middle layer pattern. (b) Topography. (c) The first mode resonance frequency of a sample with a top layer thickness of 358 nm. A PPP-FM probe was used with an applied normal force of 1162 nN.

To further demonstrate the capability of CR-AFM for imaging tiny subsurface defects in the embedded circuit pattern, we carried out CR-AFM imaging experiments on a specimen which contains well-defined defects with different dimensions. Those defects were represented by artificially introduced grooves with widths of 2, 1.8, 1.6, 1.4, 1.2, 1, 0.8, 0.7, 0.6, 0.5, 0.4, 0.3, 0.2 and 0.1 µm, respectively. A schematic illustration is sketched in Figure 9a. A sample with a cover layer thickness of approximately 358 nm was fabricated containing such defects. Then, the sample was scanned with the PPP-FM cantilever by using its first mode under an applied normal force of 1162 nN. The obtained topography and the resonance frequency image are shown in Figure 9b and Figure 9c, respectively. Even the smallest defects can be clearly observed from the CR-AFM frequency image. This indicates a spatial resolution of far better than 100 nm for CR-AFM subsurface imaging on such a sample. In fact, the subsurface lateral resolution should be closely related to several parameters and working conditions, such as contact radius, cover thickness and many others [22,45–47]. Certainly, the resolution could be far better than 100 nm.

## Conclusion

The subsurface imaging capability of CR-AFM on flexible circuits was demonstrated both experimentally and theoretically. The effects of various experimental parameters were investigated systematically. Our results show that a larger applied force can help to achieve a better contact stiffness contrast. Choosing a softer cantilever as well as using the first flexural resonance mode can also enhance the subsurface imaging on such specimens. Theoretical and FEA calculations by modelling the tip indentation on a multilayer sample agree well with the experimental results. The semi-analytical contact mechanics analysis, combined with a proper cantilever dynamics model, is proved to be convenient for guiding the experimental parameter optimization. Furthermore, detection of defects in the embedded circuit pattern was carried out by using CR-AFM, which indicates its excellent capability of imaging tiny defects smaller than 100 nm. These investigations demonstrate a nondestructive yet high spatial resolution CR-AFM method for systematic, in situ and ex situ diagnosis of flexible circuits.
